# Anti‐SARS‐CoV‐2 vaccines in recipient and/or donor before allotransplant

**DOI:** 10.1002/jha2.398

**Published:** 2022-02-17

**Authors:** Maxime Jullien, Marianne Coste‐Burel, Beatrice Clemenceau, Valentin Letailleur, Thierry Guillaume, Pierre Peterlin, Alice Garnier, Amandine Le Bourgeois, Berthe‐Marie Imbert, Jocelyn Ollier, Audrey Grain, Cyrille Touzeau, Philippe Moreau, Marie C Béné, Henri Vié, Patrice Chevallier

**Affiliations:** ^1^ Hematology Clinic Nantes University Hospital Nantes France; ^2^ Virology Department Nantes University Hospital Nantes France; ^3^ Inserm 1232, CRCINA Nantes University Nantes France; ^4^ Hematology Biology Nantes University Hospital Nantes France

**Keywords:** allogeneic, cellular response, COVID‐19, hematological, humoral response, SARS‐CoV‐2 mRNA, vaccine

## Abstract

The impact of pre‐transplant anti‐severe acute respiratory syndrome coronavirus‐2 (SARS‐CoV‐2) vaccine in 20 recipients of allogeneic hematopoietic stem cell transplantation (Allo‐HSCT) and/or their donors is reported here, showing that the persistence of anti‐SARS‐CoV‐2 antibodies can be detected in almost all patients, whatever the type of vaccine used, and up to 9 months post transplant. Also, an anti‐SARS‐CoV‐2 spike glycoprotein CD3+ T‐cell response could be detected in six (35%) of 17 evaluable patients. This study provides a rationale to consider anti‐SARS‐CoV‐2 vaccination of both recipients and donors before Allo‐HSCT.

To the editor,

Most allogeneic hematopoietic stem cell transplantation (Allo‐HSCT) recipients lose their immunity to pathogens as soon as the first months after transplant, irrespective of the pre‐transplant donor or recipient vaccinations. As such, almost all vaccines are not recommended both before the procedure, especially in donors where feasibility and ethical issues are also of concern and true benefit not proven. After a transplant, Allo‐HSCT recipients can respond to vaccines but at a lower extent than healthy individuals, and it can take months or years before detecting adequate immune response [1].

The results of anti‐severe acute respiratory syndrome coronavirus‐2 (SARS‐CoV‐2) vaccine are now progressively reported in Allo‐HSCT. Surprisingly, they show that these patients have the particularity to mount high humoral antibody responses (70%–80% of patients) when receiving the vaccination after transplant. Factors that have been associated with impaired response are ongoing systemic immunosuppressive treatment and severe lymphopenia, especially B lymphopenia. They concern a small part of the patients, explaining probably the difference in terms of immune response observed with other immunocompromised situations [2, 3, 4, 5, 6, 7, 8].

Thanks to better accessibility, more and more Allo‐HSCT‐eligible patients, as well as their related or unrelated donors, have now been vaccinated before transplant or graft collection. If coronavirus disease 2019 (COVID‐19) positive donors at the time of graft collection have been reported [9], the description and impact of such pre‐transplant vaccines are not yet reported.

In this study, anti‐SARS‐CoV‐2 antibody levels (serology 1 [S1]) and CD3+ T‐cell responses were concomitantly evaluated at a median of 68 days (range: 23–65) after Allo‐HSCT in 20 adults allografted between February 23 and July 20, 2021, in our Hematology Department, Nantes University Hospital. A second serology (S2) has been performed at a median of 168 days (range: 76–272) post‐transplant in 18 patients, including four who had received a vaccine boost before this second test.

Seven recipients vaccinated before Allo‐HSCT (R+) received a graft from a non‐vaccinated donor (D−), while five non‐vaccinated recipients (R−) received a graft from a vaccinated donor (D+). Both R/D had been vaccinated before transplant (R+/D+) in five cases. Finally, three patients who received a graft from a D− (no other donor available) had a previously COVID‐19 infection. All patients had engrafted. All (but three at S2) were under immunosuppressive drugs at S1 and S2. Details are given in Table 1. All participants gave informed consent. The study was approved by the Ethics Review Board of Nantes University Hospital.

**TABLE 1:** Patient characteristics, post‐transplant humoral and cellular anti‐S‐SARS‐CoV‐2 responses, and immune status

**Pre‐graft vaccine situation**	**Gender/age (yo)/CMV status**	**Disease**	**Conditioning/donor**	**Type of vaccine and number of doses**	**V1/I‐graft (days)**	**GVHD post‐graft**	**Graft‐S1/S2 (days)**	**Anti‐S IgG titer BAU/ml At S1/S2**	**CD4 T‐cells/mm^3^ At S1**	**CD8 T‐cells/mm^3^ At S1**	**B‐cells/mm^3^ At S1**	**NK‐ cells/mm^3^ At S1**	**γG g/L** At S1	**T‐cells Responses (SFU) At S1**
R+/D−	M/59 /−	AML	T1BF/MUD	PfizX 2	145	No	48 /76	21.2/12.9	20	45	254	377	8.5	ND
	M/71 /−	AML	Clo‐Cy‐TBI/sibling	PfizX 2	69	No	77 /98	neg/neg	197	233	0	361	5.8	0
	F/68/	AML	T1BF/MUD	PfizX 2	85	No	67 /88	9.2/6.3	0	0	11	301	9.3	5
	M/68/−	CMML	CloBalt/haplo	AZX 1	83	No	63/ 84	37.8/25.3	61	0	0	368	3.3	0
	F/66/+	ALL	CloBalt/haplo	PfizX 2	42	Yes	109/243	199/33	34	45	0	547	2.1	0
	M/47/+	MF	CloBalt/sibling	Pfiz X 2	110	Yes	23/149	>250/neg	50	235	0	387	7.9	ND
	F/67/−	AML	CloBu/MUD	PfizX 1	28	No	85/161*	4.7/1.3 **	189	93	0	107	7.4	18
R+/D+	F/69/+	Hodgkin	Balt/haplo	ModX 3	90	Yes	40/ND	>250/ND	58	45	0	237	3.9	0
	F/72/−	MDS/MPS	T1BF/MUD	PfizX 2	83	Yes	88/113	70.2/52.6	20	45	0	190	6.2	6
	M71/+	MDS	CloBalt/haplo	PfizX 2	111	No	70/84	94.8/64.7	92	314	0	422	3.8	0
	M/29/−	Hodgkin	Balt/haplo	PfizX 2	77	Yes	52/134	>250/59.7	49	140	0	82	3.9	33
	M/73/+	MF	T1BF/MUD	PfizX 2	77	No	82/134*	72.7/ > 250	50	138	0	84	5.9	36
R−/D+	M/73/+	AML	CloBalt/haplo	NA	NA	No	49/85	150/67.4	2	45	0	232	5,2	ND
	M/32/+	AML	CloBu/MUD			Yes	145/223	191/128	76	889	0	41	3,7	0
	F/42/−	Ph − ALL	CloBalt/haplo			No	23/99	160/39.4	0	123	0	23	4.4	0
	M/64/−	AML	CloBalt/MUD			No	165/272*	10/ > 250 **	143	119	97	166	9.4	35
	F/30/−	Ph+ ALL	TBI/CY/MUD			Yes	35/ND	109/ND	157	222	6	475	7,1	0
R‐but previous COVID‐19 infection/D−	M/22/−	AML	T2BF /haplo	NA	138	Yes	133/216	3.3/ > 250 **	162	91	54	114	2.6	0
	F/65/+	AML	CloBalt/haplo		192	Yes	69/140	64.3/ > 250 **	68	0	6	122	2	0
	M/59/−	Ph+ ALL	Flu‐TBI /MUD		95	Yes	63/137	>250/107	178	81	144	139	3,5	0

Abbreviations: ALL, acute lymphoblastic leukemia; AML, acute myeloid leukemia; AZ, Astra Zeneca; Balt, Baltimore regimen with fludarabine, Cy and TBI 2 grays and high‐dose post‐transplant Cy; Bu, busulfan; Clo, clofarabine; CloBalt, Baltimore regimen with clofarabine instead of fludarabine; CMML, chronic myelomonocytic leukemia; Cy, cyclophosphamide; D−, non‐vaccinated donor; D+, vaccinated donor; F, female; Flu, fludarabine; haplo, haploidentical donor; M, male; MDS, myelodysplastic syndrome; MF, myelofibrosis; Mod, Moderna.; MPN, myeloproliferative neoplasia; MUD, matched unrelated donor, NA, non‐applicable, ND, not done; Pfiz, Pfizer BioNTech; Ph, Philadelphia chromosome; R−, non‐vaccinated recipient; R+, vaccinated recipient; S1, serology post transplant; T1BF/T2BF, one or two days of thiotepa, busulfan, fludarabine; TBI, total body irradiation; V1, first dose of vaccine; yo, years old.

V1/I‐graft, days between pre‐graft first dose of the vaccine or COVID‐19 infection and graft; Graft‐S1/S2, days between graft and post‐graft serology 1 and serology 2; CD4 and CD8 T‐cells, B− and NK‐cells were evaluated at the time of post‐graft S1 and are expressed as cells/mm3; γG,: gammaglobulins were evaluated at the time of post‐graft serology and expressed as g/L.

Anti‐spike CD3+ T responses were evaluated at S1 and are expressed as a number of spot forming units (SFU) per 1 × 10^6^ CD3+ T‐cells using Interferon gamma (IFNγ) ELISpot after stimulation of immunoselected CD3+ T‐cells with 3 SARS‐CoV‐2 spike proteins.

* Off of immunosuppressive drugs.

** Boost vaccine between S1 and S2.

Immunoglobulin G (IgG) to the SARS‐CoV‐2 spike protein receptor‐binding domain was assayed (Roche Elecsys) with titers ≥ 0.8 BAU/ml considered positive, the highest threshold being > 250 BAU/ml. Remarkably, the persistence of anti‐SARS‐CoV‐2 antibodies can be detected in 95% of patients (*n* = 19/20) at S1 and 89% of patients (*n* = 16/18) at S2, whatever the type of vaccine used, until 9 months post transplant. Only one R+/D− case showed seronegativity at S1 and S2. The median positive IgG titer for the whole group was 83.75 BAU/ml (0 → 250, the latter for four patients) at S1 and 128 BAU/ml (0 → 250, the latter for four patients) at S2. Median IgG titers at S1 and S2 differed between R+/D− (21.2 and 6.3), R−/D+ (150 and 189, and R+/D+ (94.8 and 128) and infected R/D− (64.3 and > 250) groups. Positivity in R−/D+ and R+/D− together with infected/D− patients indicates anti‐S‐SAR‐CoV‐2 antibodies from donor and recipient origins, respectively. Also, between S1 and S2, IgG titers decreased in all patients, except in four including three who had received a boost vaccine after S1. Positivity was obtained even with no peripheral B‐cells, but a median 3.9 g/L level of gammaglobulins (without supplementation) suggests the persistence of pre‐transplant antibodies or germinal center B‐cell responses [10].

Anti‐COVID‐19 T‐cell response analysis could be performed at S1 in 17 patients and 16 healthy controls. IFNγ ELISpot (Human ELISpo Kit, Mabtech 3420–2AST‐10) were performed with 60,000 to 200,000 immuno‐selected CD3+ T‐cells stimulated with culture medium (negative control), three peptide pools covering the whole protein sequence of the SARS‐CoV‐2 spike glycoprotein (Prot _S1; _S+ and _S PepTivator peptide pools, Miltenyi Biotec) or Ebstein‐Barr Virus (EBV)‐consensus peptides (EBV peptivator consensus, Miltenyi Biotec, positive control). Anti CD4+ or CD8+ T‐cell responses analyses could not be performed (except in one case) because of insufficient material.

All fully vaccinated healthy donors developed positive T‐cell responses to spike peptide pools even though variable frequencies were observed. The median response was 195 SFU/106 CD3+ T‐cells, which corresponds to a frequency of 0.02%. Anti‐SARS‐CoV‐2 spike glycoprotein CD3+ T‐cell response could be detected in six (35%) of 17 evaluable patients, including 2/5 (20%) R+/D− cases, 3/5 (60%) R+/D+ cases (one showing also anti‐spike CD8+ T‐cell response), 0/3 patients with previous COVID‐19 infection, and 1/4 (25%) R−/D+ cases. However, the frequencies of specific anti‐SARS‐CoV‐2 T‐cells for these six patients were almost eight times lower than for vaccinated healthy donors. The median response was 26 SFU/106 CD3+ T‐cells, which corresponds to a frequency of 0.003%. T‐cell responses were null in the three patients previously infected probably because they were all on immunosuppressive drugs at the time of evaluation, especially under corticosteroids for graft‐versus‐host disease (GVHD). However, two of these patients obtained the maximal IgG titer after receiving a boost vaccine, suggesting that patients with previous COVID‐19 infection could respond optimally after the boost after the graft. The third patient has not yet received this boost. Results are shown in Table 1 and Figure 1.

**FIGURE 1:**
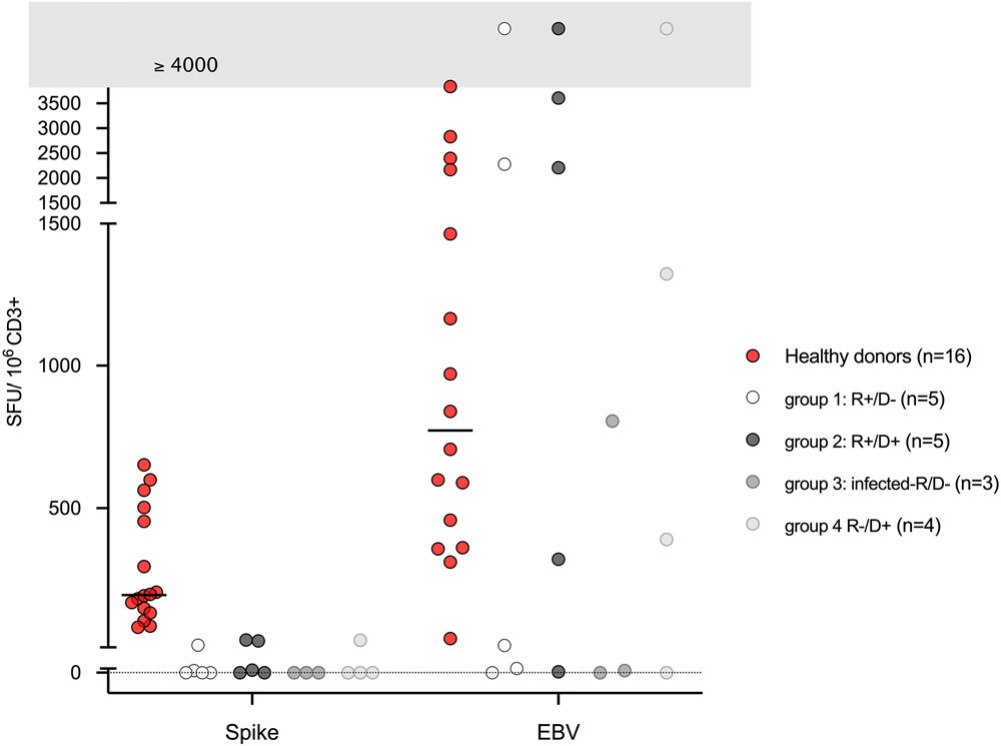
Anti‐SARS‐CoV‐2 sike and anti‐EBV T‐cells analysis in allogeneic hematopoietic stem cell transplantation (Allo‐HSCT) patients (*n *= 16) and healthy donors (*n* = 16) after the second injection of mRNA COVID‐19 vaccine (BNT162b2, Pfizer‐BioNTech). The interval between the second dose and the T‐cell response analysis was 56 and 55 days, respectively, for healthy donors and Allo‐HSCT patients. IFNγ ELISpot were performed with 60,000 to 200,000 immunoselected CD3+ T‐cells stimulated with spike, EBV‐consensus peptides (Miltenyi) or medium (as negative control). The number of spot forming units (SFU) obtained in the stimulated condition was subtracted from that obtained in the unstimulated condition (medium alone). Results showed the number of SFU per 1 × 106 CD3+ T‐cells. For healthy donors, bars represent median

Only one patient (with both S1 and S2 negative serology) had developed (severe) COVID‐19 infection at the last follow‐up (January 2022).

In contrast with current recommendations [1], this study provides a rationale to consider anti‐SARS‐CoV‐2 vaccination of both R/D before Allo‐HSCT. It remains to determine whether these antibodies and T‐cell response provide sufficient protection after transplant. A threshold of an IgG titer of 250 BAU/ml has been associated with an estimate of close to 90% of mRNA‐1273 efficacy in the COVE trial [11]. Based on this trial, detectable antibody responses can be classified as “weak” in the case of < 250 BAU/ml and as “good” in the case of ≥ 250 BAU/ml [12]. Here, a good titer has been detectable only in four patients after transplant, suggesting that one or more post‐transplant booster shots are needed as already reported before the era of pre‐transplant vaccine [13, 14].

## CONFLICT OF INTEREST

The authors declare no conflict of interest.

## AUTHOR CONTRIBUTION

Maxime Jullien and Patrice Chevallier designed, performed, coordinated the research, analyzed and interpreted the data, generated the table, and wrote the manuscript. Marianne Coste‐Burel performed serology tests, generated virologic data and commented on the manuscript. Beatrice Clemenceau, Jocelyn Ollier, and Audrey Grain performed cellular analyses, generated the figure and commented on the article.

## Data Availability

All authors had full access to all the data in the study and take responsibility for the integrity of the data and the accuracy of the data analysis. All data generated or analysed during this study are included in this published article.
